# Inflammation-Associated Changes in Bioactive Proteins and Peptides of Bovine Milk: Evidence from Mastitis, Lameness, and Metabolic Disorders—A Review

**DOI:** 10.3390/molecules31152570

**Published:** 2026-07-23

**Authors:** Levente Kovács, Lilla Sándorová, Ferenc Pajor

**Affiliations:** 1 Department of Animal Husbandry and Animal Welfare, Institute of Animal Sciences, Hungarian University of Agriculture and Life Sciences, Páter Károly utca 1, H-2100 Godollo, Hungary; pajor.ferenc@uni-mate.hu; 2 Bona Adventure Ltd., Peres utca 44, H-2100 Godollo, Hungary; 3 Department of Genetics and Genomics, Institute of Genetics and Biotechnology, Hungarian University of Agriculture and Life Sciences, H-2100 Godollo, Hungary; sandorova.lilla@uni-mate.hu

**Keywords:** bovine milk, bioactive peptides, acute-phase proteins, lactoferrin, mastitis, lameness, metabolic disorders, milk proteomics, dairy processing

## Abstract

Bovine milk contains bioactive proteins and encrypted peptide sequences whose abundance and availability may change during mammary or systemic inflammation. This narrative review critically evaluates evidence associated with subclinical mastitis, lameness-causing claw disorders, and periparturient metabolic disorders. Direct milk-level evidence is strongest for mastitis: increased somatic cell count and intramammary inflammation are generally associated with higher concentrations of milk haptoglobin, milk serum amyloid A (including mammary-associated serum amyloid A3, when isoform-resolved), lactoferrin, cathelicidins, and immunoglobulins, together with accelerated casein proteolysis. For lameness and metabolic disorders, evidence is substantially weaker and derives mainly from systemic acute-phase responses, broad milk metabolomic or compositional changes, and mechanistic hypotheses involving blood–milk barrier permeability and protease regulation. The review explicitly separates direct disease-defined milk evidence from systemic, broad omics, and mechanistic evidence. These alterations may influence biomarker performance, whereas processing consequences are best-established for mastitic milk. Their persistence after processing and digestion, and their biological effects in human consumers, remain largely unresolved. Future studies should combine diagnosis-specific cow health monitoring with milk proteomics and peptidomics. They should also assess the stability, bioaccessibility, and functionality of altered proteins and peptides during processing and digestion.

## 1. Introduction

Bovine milk is a complex biological fluid containing intact bioactive proteins and peptide sequences encrypted within caseins and whey proteins. Lactoferrin, immunoglobulins, lysozyme, cathelicidins, acute-phase proteins (APPs), and milk-protein-derived peptides have demonstrated antimicrobial, immunomodulatory, antioxidant, antihypertensive, or opioid activities, primarily in biochemical, cellular, or digestion-based experimental systems [1,2,3,4,5,6,7,8,9]. Their relevance in dairy foods and to consumers depends on concentration, structural integrity, processing stability, digestive release, and bioavailability [7,10,11,12,13].

The inflammatory status of the lactating cow can alter this fraction through altered mammary secretion, paracellular transfer across a disrupted blood–milk barrier, and activation of endogenous or leukocyte-derived proteases [14,15,16,17,18,19,20,21]. These processes can increase immune proteins while reducing casein integrity and changing substrates available for processing and digestion. This review therefore focuses on disease-associated changes in milk bioactive proteins and peptides rather than on inflammation in dairy cows generally.

Mastitis provides the clearest disease model because inflammation occurs within the mammary gland, and affected quarters can be sampled directly. Subclinical mastitis (SCM) is defined here as a somatic cell count (SCC) > 200,000 cells/mL without visible clinical signs [16,22,23]. Lower cut-offs of approximately 100,000–150,000 cells/mL are also used for sensitive quarter-level screening [22,23], whereas European Union legislation specifies a rolling geometric mean of ≤400,000 cells/mL over three months for raw bulk cow milk [24]. Comparisons should therefore report the sampling unit, parity, repeated-sampling scheme, pathogen status, and diagnostic objective.

Lameness-causing claw lesions can elicit local and systemic inflammation, but disease-specific measurements of milk bioactive proteins and peptides remain scarce [25,26,27]. Periparturient negative energy balance, hyperketonemia, hypocalcemia, and hepatic lipidosis can alter immune function, nutrient partitioning, and potentially milk-protein synthesis or protease regulation [28,29,30,31,32,33,34,35,36,37,38,39,40,41]. Because these conditions often overlap and production level varies with breed, parity, diet, region, and management, this review applies no universal milk-yield threshold for high-producing cows [42].

The evidence base is therefore uneven: mastitis is supported by direct milk measurements and challenge studies, whereas conclusions for lameness and metabolic disease rely largely on systemic biomarkers, broad compositional changes, or mechanistic inference. This distinction matters for health monitoring, dairy processing, and consumer relevance, the last of which additionally requires persistence through processing and digestion and biological activity at physiologically relevant exposure levels [7,10,11,12,13,43,44].

**Figure 1:**
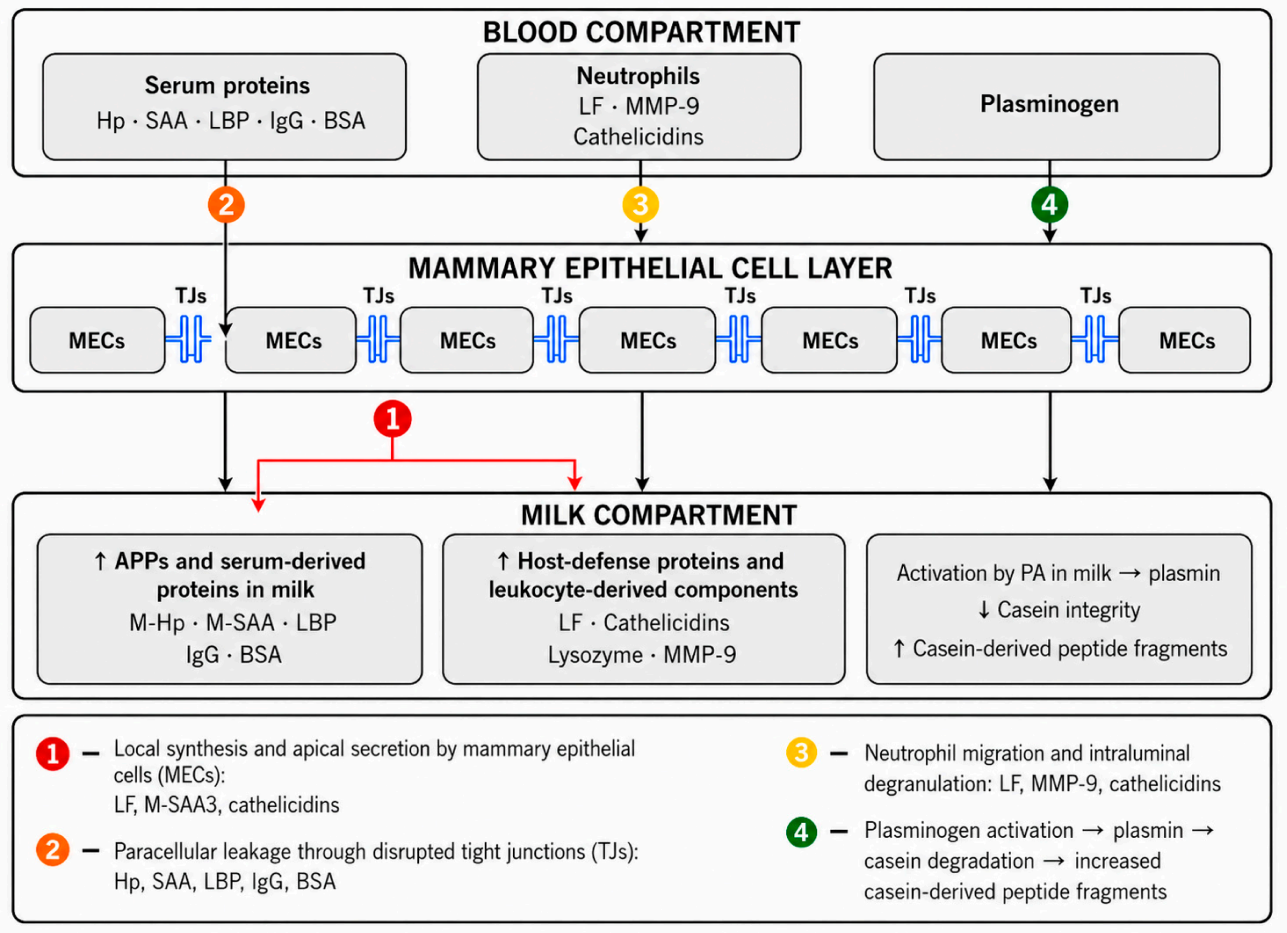
Principal pathways by which subclinical inflammation may alter the bioactive protein and peptide fraction of bovine milk. Four related mechanisms are illustrated: (i) local synthesis and apical secretion by mammary epithelial cells (MECs), contributing lactoferrin, cathelicidins, and mammary-associated serum amyloid A3 (M-SAA3) to milk; (ii) paracellular transfer through inflammation-disrupted tight junctions (TJs), allowing circulating proteins, including haptoglobin, serum amyloid A, lipopolysaccharide-binding protein, immunoglobulins, and bovine serum albumin, to enter milk; (iii) neutrophil migration into the mammary lumen and subsequent degranulation, releasing lactoferrin, matrix metalloproteinase-9, cathelicidins, and other leukocyte-derived components; and (iv) activation of milk plasminogen by plasminogen activators, generating plasmin and promoting casein degradation and the release of casein-derived peptide fragments. These pathways may occur simultaneously, although their relative contributions vary with the cause, location, and intensity of inflammation. The schematic represents pathways relevant to subclinical inflammatory conditions and is not intended to quantify changes occurring during overt clinical disease. Arrows indicate the direction of the proposed pathways or changes; ↑ and ↓ denote increases and decreases, respectively. APPs = acute-phase proteins; BSA = bovine serum albumin; Hp = haptoglobin; IgG = immunoglobulin G; LBP = lipopolysaccharide-binding protein; LF = lactoferrin; MEC = mammary epithelial cell; M-Hp = milk haptoglobin; MMP-9 = matrix metalloproteinase-9; M-SAA = milk serum amyloid A when the assay does not resolve the isoform; M-SAA3 = mammary-associated serum amyloid A3; PA = plasminogen activator; SAA = serum amyloid A; TJ = tight junction [3,18,20,45,46].

Figure 1 summarizes the principal routes by which inflammation may modify the milk bioactive fraction. Active secretion, serum-protein leakage, neutrophil migration, and protease activation can occur simultaneously, although their relative contributions differ between intramammary and systemic disease [14,15,16,17,18,19,20,21]. The schematic represents subclinical pathways rather than a quantitative model of overt clinical inflammation.

This review integrates evidence across mastitis, claw disorders, and periparturient metabolic disease while explicitly classifying the directness and endpoint specificity of the available evidence. It aims to summarize direct changes in milk bioactive proteins and peptides during intramammary infection and SCM; evaluate comparable findings in lameness and defined claw lesions; examine ketosis and other metabolic disorders in relation to milk protein integrity, APPs, and peptide-generating proteases; and assess the fate and possible functionality of these changes during processing and digestion without extrapolating raw-milk findings directly to consumer health.

Established milk-level findings are separated from indirect systemic evidence and mechanistic hypotheses. This evidence-aware structure supports a One Health perspective [47] without assuming that a change detected in the cow, raw milk, or an in vitro assay necessarily affects a processed dairy product or the human consumer.

## 2. Methods

This article is a critical narrative review designed to evaluate the directness and endpoint specificity of evidence linking mastitis, lameness-causing claw disorders, and periparturient metabolic disorders with changes in bovine milk bioactive proteins, proteolytic systems, casein integrity, and peptide-related endpoints.

Relevant literature was identified through iterative, targeted searches of PubMed/MEDLINE, Web of Science, Scopus, and CAB Abstracts. Search concepts covered three principal domains: (i) bovine milk bioactive proteins, proteases, and peptides; (ii) mastitis, lameness and defined claw lesions, and periparturient metabolic disorders; and (iii) milk quality, dairy processing, gastrointestinal digestion, and potential human-health relevance. Searches were supplemented by backward citation screening of relevant primary studies and reviews. No publication-date restriction was applied. The literature searches were last updated on 15 June 2026.

For the disease-focused synthesis, peer-reviewed English-language publications were considered when they reported milk proteins, protease activity, casein integrity, defined peptide endpoints, or broad milk-omics or compositional findings in cows with a defined diagnosis, lesion classification, or experimental mammary challenge. Serum, plasma, tissue-level, inflammatory, or physiological studies were included only as indirect contextual evidence and were not interpreted as demonstrating a corresponding change in milk. Studies on processing, gastrointestinal digestion, purified proteins or peptides, experimental animals, or human biological effects were used only to evaluate downstream stability, bioaccessibility, or functionality. Regulatory documents, guidelines, and selected books or book chapters were used separately for definitions, diagnostic thresholds, analytical context, and regulatory interpretation.

Evidence directness and endpoint specificity were classified at the level of each disease–endpoint relationship using an A–D framework. Category A denotes direct milk-level evidence for the specified protein, protease, peptide, or explicitly defined milk endpoint from cows with a defined diagnosis or from an experimental mammary challenge. Category B denotes relevant evidence that does not directly quantify the specified endpoint and is further described as B—systemic, referring to serum, plasma, tissue-level, inflammatory, or physiological evidence, or B—milk-omics, referring to direct but broad milk metabolomic, lipidomic, proteomic, or compositional measurements that do not quantify the specified bioactive protein, protease, or peptide endpoint. Category C was assigned when a specific mechanistic relationship between a disease process and the specified milk endpoint was supported by relevant indirect or non-disease-specific evidence but still required disease-specific milk validation. Category D was assigned when neither direct disease-specific milk evidence nor a sufficiently specific, evidence-supported mechanistic relationship was identified among the sources considered. Category D therefore indicates an evidence gap rather than evidence that no change occurs.

Where different evidence types were available for the same disease–endpoint relationship, the classification reflected the most direct evidence identified, while analytical limitations, inconsistencies, and important confounders were described in the accompanying text. Mechanistic plausibility alone was not used to classify an endpoint as direct milk-level evidence. Analytical approaches were also considered during interpretation because immunoassays, activity assays, electrophoresis, and mass spectrometry-based methods differ in sensitivity, specificity, calibration, sample preparation, and analyte coverage.

The A–D framework describes evidence directness and endpoint specificity only and does not constitute a formal assessment of study quality, risk of bias, consistency, reproducibility, or overall certainty. Category A should therefore not automatically be interpreted as high-quality or high-certainty evidence, and category B should not be interpreted as methodologically inferior evidence.

Because the searches were iterative and were not prospectively documented as a formal systematic-review workflow, database-specific retrieval counts, complete pre-deduplication records, and a formal screening log were not retained and could not be reconstructed retrospectively with sufficient reliability. These numbers are consequently not reported. The final reference list contains 101 unique disease-focused, methodological, regulatory, and contextual sources cited in the manuscript. This number represents the total number of cited sources and should not be interpreted as the number of studies selected through a formal systematic-review screening process. No formal duplicate independent screening, risk-of-bias assessment, or quantitative synthesis was undertaken. The restriction to English-language disease-focused publications may have introduced language bias because relevant dairy-cattle research is also available in German, French, Hungarian, and other languages.

## 3. Bioactive Proteins and Peptides in Healthy Bovine Milk

### 3.1. Major Bioactive Proteins

The bioactive protein fraction of bovine milk includes molecules involved in mammary defense, neonatal immunity, and milk functionality. Lactoferrin (LF), an iron-binding glycoprotein, is reported at approximately 0.03–0.5 mg/mL in healthy mature individual-cow or quarter milk, with substantial variation according to lactation stage, SCC, sampling design, and analytical method [3,48]. It increases during mammary inflammation. It restricts microbial growth by sequestering iron and by interacting with bacterial surfaces and lipopolysaccharide; proteolysis can also release membrane-disrupting peptides such as lactoferricin [3,43]. Interactions with viral or host-cell surfaces and the modulation of cytokine and leukocyte activity provide mechanisms for its antiviral, anti-inflammatory, and immunomodulatory effects [3].

Other bioactive proteins include immunoglobulins, lysozyme, cathelicidins, and acute-phase proteins (APPs). In healthy mature quarter milk, representative values are approximately 0.2–0.5 mg/mL for IgG1 or total IgG, although the reported concentration depends strongly on isotype, lactation stage, SCC, sampling unit, and assay [2,8,48]. By contrast, milk haptoglobin (M-Hp) and mammary-associated serum amyloid A3 (M-SAA3) are commonly low, undetectable, or near the assay-specific detection limit in healthy control milk in the cited experimental studies; the available data do not establish universal healthy-milk reference intervals [14,15]. Lysozyme and cathelicidins provide antimicrobial defense [16,49], while lipopolysaccharide-binding protein and α1-acid glycoprotein are often near assay quantification limits [16,19]. Throughout this review, M-SAA3 denotes the mammary-associated isoform, whereas M-SAA denotes milk serum amyloid A when the cited assay or report does not resolve the isoform.

Reported concentrations vary with lactation stage, parity, breed, udder-health definition, sampling level, and analytical platform. Values also differ substantially according to whether total IgG or a specific isotype is measured and whether quarter, composite, or bulk milk is analyzed. Terms such as “low”, “undetectable”, or “near the limit of quantification” therefore describe assay-dependent observations rather than biological absence. Table 1 provides representative values or assay-status descriptions, together with detection approaches reported in the cited literature. The method column summarizes approaches used for each analyte and does not imply that every cited study used every listed platform; study-specific protocols should be checked in the original reports.

### 3.2. Bioactive Peptides Derived from Milk Proteins

Bioactive sequences encrypted within caseins and whey proteins require proteolytic release during digestion, fermentation, industrial hydrolysis, or endogenous milk proteolysis [4,5,6,7,44]. Casein-derived products include the ACE-inhibitory peptides Val-Pro-Pro (VPP) and Ile-Pro-Pro (IPP), β-casomorphins, and immunomodulatory or antibacterial sequences; whey proteins also yield antimicrobial, antioxidant, and immunomodulatory peptides [4,5,6,7].

The presence of a sequence in an intact parent protein does not demonstrate a measurable free peptide in raw milk or predict the amount released during processing or digestion. Table 1 therefore treats ACE-inhibitory peptides and β-casomorphin-7 (BCM-7) as peptide-related endpoints. Targeted LC–MS/MS and sequence-resolved peptidomics quantify individual peptides, whereas ACE-inhibition assays measure the combined activity of a peptide mixture; these endpoints are not interchangeable [7].

Peptide generation and persistence depend on the extent and specificity of proteolysis. Plasmin preferentially cleaves β-casein and produces γ-caseins and proteose-peptone fragments [17,21,54]; its activity reflects the balance among plasminogen, plasmin, activators, and inhibitors and increases during mammary inflammation and late lactation [17,21]. Mastitis clearly increases plasmin activity and reduces casein integrity, but sequence-resolved changes in VPP, IPP, and other defined peptides remain insufficiently documented. Metabolic-disease-related claims concerning VPP and IPP are likewise classified as category C because altered casein integrity or protease regulation could affect their subsequent release during processing or digestion, although no disease-specific sequence-resolved evidence is currently available [4,5,21,54,58,59].

BCM-7 illustrates the distinction between substrate composition and demonstrated exposure. It can be released from A1 and B β-casein during enzymatic or gastrointestinal digestion, whereas release from A2 β-casein is more restricted [60,61,62]; the A1:A2 ratio is determined mainly by herd CSN2 genotypes [61]. Inflammation may modify β-casein integrity, but no disease-defined study has shown a reproducible effect on free BCM-7 in raw milk or its subsequent generation. Mastitis- and metabolic-disease-related BCM-7 claims therefore remain category C under the evidence-directness framework [21,54,58,59,60,61,62]. Lameness-related claims remain category D.

## 4. Effects of Mastitis on the Bioactive Protein and Peptide Profile of Milk

### 4.1. Intramammary Infection and Acute-Phase Protein Response

Evidence-directness summary: A for direct, endpoint-specific mastitis-associated changes in milk acute-phase and host-defense proteins.

Mastitis is commonly associated with bacterial intramammary infection, including *Staphylococcus aureus*, coagulase-negative staphylococci, *Escherichia coli*, and *Streptococcus* spp. Mammary epithelial activation, neutrophil recruitment, increased SCC, blood–milk barrier disruption, and local TNF-α, IL-1β, and IL-6 production accompany infection [18,63]. Experimental intramammary LPS challenge increased milk haptoglobin [14]. Experimentally induced *S. aureus* mastitis increased milk haptoglobin and serum amyloid A [15]. Clinical and systematic evidence supports these proteins as adjunct biomarkers, although performance varies with severity, bacteriological status, sampling design, and analytical method [16,50,51].

Milk LF and cathelicidins also increase during intramammary inflammation. LF reflects mammary epithelial secretion and neutrophil recruitment, with responses influenced by lactation stage, SCC, pathogen category, and assay [3,16,48]. Cathelicidins contribute to local antimicrobial defense and have potential as subclinical and clinical mastitis biomarkers [16,49,50]. These are direct milk-level findings, but their individual technological effects should be distinguished from the better-established consequences of mastitis-associated proteolysis.

Milk APP responses vary among pathogens, although Gram-negative and Gram-positive categories overlap. Gram-negative or lipopolysaccharide challenges often induce rapid, pronounced responses, while *S. aureus* and some coagulase-negative staphylococci may produce lower, persistent patterns [14,15,18,22]. In natural mastitis, M-Hp, M-SAA, and cathelicidin also vary with bacteriology, clinical severity, and systemic inflammation [50,51]; a single APP threshold is therefore unlikely to perform uniformly across etiologies and lactation stages.

Diagnostic systems should combine repeated APP measurements with SCC, days in milk, parity, clinical assessment, and pathogen identification. Multi-analyte panels incorporating M-Hp, M-SAA, cathelicidins, LF, and other inflammatory proteins may be more robust than a single marker, but require external validation across farms, platforms, and pathogen groups [16,50]. Proteomic studies have identified additional host-response candidates [19,49,64], which must be validated by standardized quantitative assays before routine use.

These findings establish altered APP and host-defense protein concentrations in raw mastitic milk, not their retention or activity after processing and digestion or any effect in human consumers. Those evidence tiers are considered separately in Section 7 [7,10,11,12,13].

### 4.2. Effects on Casein Integrity and Bioactive Peptide Availability

Evidence-directness summary: A for direct, endpoint-specific evidence of mastitis-associated activation of milk proteolytic systems and loss of casein integrity, and C for disease-specific changes in defined bioactive peptide sequences and their biological functionality.

Mastitis activates several milk proteolytic systems. Plasmin is the principal endogenous caseinolytic enzyme, while leukocytes and mammary cells contribute matrix metalloproteinases (MMPs) and other proteases. Experimental and disease-defined studies show increased proteolytic or gelatinolytic activity, accelerated casein degradation, and increased MMP-9 activity, with less-consistent MMP-2 responses [21,54,55,56,57]. These data demonstrate reduced casein integrity but not the generation or persistence of specific bioactive peptide sequences.

In a preclinical study, VPP and IPP reduced lipopolysaccharide-induced inflammatory responses in cultured bovine mammary epithelial cells and showed protective effects in an LPS-induced mouse mastitis model [65]. These results demonstrate activity under experimental conditions but not endogenous generation at effective concentrations or an in vivo feedback effect in dairy cows. The proposed relationship therefore remains a category C hypothesis under the evidence-directness framework.

MMP-2 and MMP-9 act mainly on extracellular-matrix and basement-membrane substrates, whereas plasmin preferentially hydrolyses caseins, especially β-casein. Gelatinases can facilitate leukocyte migration and barrier damage [18,56,57], thereby modifying whey-protein transfer [18,20]. Because mastitic milk contains mammary-, leukocyte-, serum-, and potentially pathogen-derived proteases, total proteolytic activity cannot be assigned to a single enzyme [21,54,55,57], and MMP-2 and MMP-9 need not change in parallel [56,57].

Mastitis-associated proteolysis prolongs rennet coagulation, weakens gels, and impairs cheese making by reducing intact α- and β-casein and altering micelle properties [21,54,55,66]. Effects depend on pathogen, inflammatory severity, SCC, lactation stage, initial casein composition, and the proportion of affected milk in the bulk tank [55,66,67]. Although peptide fragments and proteose-peptone fractions may increase, sequence-resolved evidence remains too limited to infer a reproducible or beneficial bioactive peptide profile [55].

### 4.3. Subclinical Mastitis: Magnitude of Changes

Evidence-directness summary: A for direct, endpoint-specific subclinical-mastitis-associated changes in milk acute-phase and host-defense proteins, although the magnitude and diagnostic performance of individual markers vary with pathogen, sampling design, lactation stage, and analytical method.

SCM is defined here as SCC > 200,000 cells/mL without visible udder or milk abnormalities [16,22,23]. Lower thresholds of approximately 100,000–150,000 cells/mL may be used for sensitive quarter-level screening, while the European Union bulk-milk criterion is a rolling geometric mean of ≤400,000 cells/mL over three months [24]. Studies should report the sampling level, single or repeated measurement, and bacteriological or molecular confirmation because these choices affect prevalence, effect size, and diagnostic performance.

Direct milk studies show that SCM can increase M-Hp, M-SAA, cathelicidins, and sometimes LF [16,50,53], while altering serum-derived proteins, casein fractions, and protease activity [53,56]. The magnitude is heterogeneous because pathogens, duration, inflammatory intensity, SCC, and lactation stage differ among cows and studies; elevated SCC therefore does not imply a uniform response in every bioactive protein.

Pathogen-defined SCM studies have identified host-defense and casein-associated changes. *Streptococcus agalactiae*-associated SCM was linked to an altered protein profile and cathelicidin-1 abundance was associated with SCC [53], and naturally occurring SCM has been associated with altered composition and MMP-9 activity [56]. Clinical mastitis and experimental *E. coli* proteomic studies [19,64] provide mechanistic context but not direct estimates of changes in natural SCM.

Biomarker performance is assay- and study-specific. In one quarter-level study, haptoglobin showed sensitivity 0.92 and specificity 0.94, cathelicidin 0.83 and 0.97, and M-SAA 0.65 and 0.76, respectively [50]. Haptoglobin and cathelicidin also varied with bacteriological findings [50], indicating that optimal thresholds may differ among pathogens.

Pathogen dependence complicates universal mastitis detection: thresholds suited to strong neutrophilic responses may miss persistent low-grade infections, while lower thresholds may lose specificity in late lactation, systemic inflammation, or bacteriologically negative high-SCC quarters. Studies should report pathogen distribution, days in milk, parity, sampling level, assay platform, and the SCM reference definition. Repeated measurements interpreted with SCC and microbiology are more defensible than a universal cut-off [16,50].

Multi-analyte panels may improve discrimination, but superiority over individual markers has not been validated consistently across independent herds and platforms. Prospective external validation, standardized assays, repeatability testing, and clearly defined quarter-level infection status are required before use in automated milking systems. Bulk-tank SCC can dilute signals from a few affected quarters, so specific protein biomarkers should be validated at cow or quarter level [16,23,50].

SCM studies establish altered raw-milk protein concentrations and profiles, not structural retention after processing and digestion or beneficial or adverse effects in consumers. These later evidence levels are addressed in Section 7.

## 5. Effects of Lameness and Claw Disorders on Milk Bioactive Components

### 5.1. Systemic Inflammatory Responses Associated with Lameness-Causing Claw Disorders

Evidence-directness summary: B—systemic for lesion-associated systemic inflammatory responses and C–D for disease-specific changes in milk bioactive proteins and peptides.

Lameness is a clinical sign with diverse causes, most commonly digital dermatitis, sole ulcer, white line disease, heel horn erosion, and laminitis [25,68]. Lesions differ in etiology and inflammatory intensity. Serum Hp and SAA can increase with claw pathology [26,52]; acute laminitis and sole ulcer produced the largest responses in one lesion-specific study, digital dermatitis a moderate response, and heel horn erosion or white line separation no significant change [52]. These findings demonstrate systemic inflammation in selected lesions, not corresponding milk changes.

Longitudinal studies have detected altered circulating innate-immunity reactants before clinical lameness [27]. However, transition-period physiology, negative energy balance, concurrent metabolic disease, and later claw lesions may contribute simultaneously. Such data therefore support an association with systemic inflammation, not a direct effect of an isolated lesion on the milk bioactive profile.

Most evidence derives from serum, plasma, or claw tissue. Hp and SAA are nonspecific and also increase with mammary, uterine, respiratory, gastrointestinal, and metabolic disease [69]. Systemic APP elevation in a lame cow cannot therefore be assumed to increase milk Hp, M-SAA, LF, cathelicidins, or peptides, and any milk change requires the exclusion of intramammary and other inflammatory disease. Proposed links through barrier permeability, serum transfer, nutrient partitioning, or mammary proteases remain mechanistic hypotheses.

Disease-specific milk studies are scarce. Stronger designs should combine standardized lesion classification with repeated quarter-level SCC, culture or PCR, exclusion of current or recent mastitis, and adjustment for days in milk, parity, yield, body condition, feed intake, blood β-hydroxybutyrate (BHBA), non-esterified fatty acids (NEFAs), medication, and postpartum disease. Longitudinal serum and milk sampling before diagnosis, at detection, after treatment, and during recovery, together with targeted protein, casein, plasmin, proteomic, and peptidomic measurements, would test whether a reproducible claw-disease-specific milk signature exists. Table 2 summarizes the available lesion-level evidence.

### 5.2. Milk Metabolomic and Lipidomic Evidence in Lame Cows

Evidence-directness summary: B—milk-omics for broad metabolomic and lipidomic measurements in milk from selected lameness-classified cohorts, because these studies do not directly quantify the specified bioactive protein or peptide endpoints; C–D for lesion-specific changes in bioactive proteins, protease systems, or defined peptide sequences.

Direct milk-level omics studies demonstrate broad signatures, but they do not provide endpoint-specific evidence for the bioactive proteins and peptides reviewed here. Untargeted dried-milk-spot analysis distinguished lame from non-lame cows using lipid and small-metabolite signals, with phosphatidylglycerol (PG) 35:4 identified as a candidate marker based on internal validation [70]. First-lactation milk lipidomics also identified signals associated with subsequent lameness and onset [71]. These studies demonstrate broad metabolomic or lipidomic differences, not changes in APPs, LF, cathelicidins, casein-derived peptides, or other defined protein endpoints.

Most milk-omics studies classified cows by mobility or later lameness status without consistently identifying the responsible lesion or excluding SCM. Signatures may therefore reflect pain, reduced intake, energy balance, lactation stage, medication, yield, or concurrent disease. A scoping review likewise found that most lameness biomarker studies examined serum or plasma rather than milk [72].

Available claw-lesion studies measured APPs in serum rather than milk [26]. They support lesion-associated systemic inflammation but not increased milk haptoglobin. Distinguishing a systemic claw effect from secondary mammary inflammation requires lesion-specific diagnosis, repeated quarter-level SCC, culture or PCR, exclusion of current or recent mastitis, and simultaneous serum and milk sampling.

Days in milk, parity, yield, body condition, feed intake, BHBA, NEFA, medication, and postpartum disorders should be controlled by matching, exclusion, or multivariable analysis. Longitudinal sampling and targeted assays for M-Hp, M-SAA, LF, cathelicidins, casein integrity, plasmin, and sequence-resolved proteomics or peptidomics would provide stronger causal evidence.

Lameness-related pain and behavioral change can reduce feeding and milk production [73,74,75], but these studies do not demonstrate altered casein-to-whey ratio, proteolysis, or a peptide profile. Production, metabolomic, and lipidomic changes should therefore be distinguished from targeted evidence for bioactive proteins and peptides.

**Table 2:** Systemic acute-phase responses associated with selected claw disorders and availability of direct milk-level evidence.

Claw Disorder	Systemic Inflammatory Evidence	Sample Matrix and Principal Endpoints	Direct Milk Bioactive Evidence	Evidence-Directness Category	References
Digital dermatitis	Serum Hp and SAA increases have been reported, but responses are generally lower and less consistent than for sole ulcer or acute laminitis	Serum; Hp and SAA immunoassays	No lesion-specific milk APP, proteomic, or peptidomic study identified	B—systemic (serum); D for specified lesion-specific milk endpoints (evidence gap).	[52,76]
Sole ulcer	Consistent systemic inflammatory response; elevated SAA may persist for at least two weeks after diagnosis or treatment	Serum; Hp, SAA, IL-6, and clinical inflammatory measures	No direct milk APP or bioactive protein measurement in mastitis-free cows identified	B—systemic (serum); D for specified lesion-specific milk endpoints (evidence gap).	[26,52,76]
White line disease/separation	Serum APP responses are low or inconsistent and have not differed from controls in some studies	Serum; Hp and SAA	No direct milk data identified	B—systemic (serum); D for specified lesion-specific milk endpoints (evidence gap).	[52,76]
Acute laminitis	Marked serum Hp and SAA increases reported in a lesion-specific cross-sectional study	Serum; Hp and SAA ELISA	No direct milk data identified	B—systemic (serum); D for specified lesion-specific milk endpoints (evidence gap).	[52]
Heel horn erosion	Minimal or no significant serum Hp and SAA response relative to controls in the available lesion-specific study	Serum; Hp and SAA ELISA	No direct milk data identified	B—systemic (limited serum evidence); D for specified lesion-specific milk endpoints (evidence gap).	[52]

Note: Hp and SAA findings are derived from serum measurements and should not be interpreted as evidence that the corresponding proteins increase in milk. Study-to-study comparisons are limited by differences in lesion definition, severity, sampling time, treatment status, analytical method, lactation stage, and control of concurrent disease. No study identified in this review simultaneously confirmed a specific claw lesion, excluded intramammary infection using repeated quarter-level testing, and quantified the corresponding milk APP, proteomic, or peptidomic response. APP = acute-phase protein; ELISA = enzyme-linked immunosorbent assay; Hp = haptoglobin; IL-6 = interleukin-6; SAA = serum amyloid A.

### 5.3. Implications for the Milk Bioactive Protein and Peptide Fraction

Evidence-directness summary: C for a possible relationship between claw disease and the milk bioactive fraction, and D for lesion-specific changes in individual milk bioactive proteins or defined peptide sequences.

Current evidence does not show that isolated lameness or a specific claw lesion reproducibly changes milk haptoglobin, M-SAA, LF, cathelicidins, plasmin, casein integrity, or defined peptides. Broad milk metabolomic and lipidomic differences [70,71] should not be interpreted as altered bioactive protein or peptide availability, and no direct evidence supports increased LF or cathelicidins in mastitis-free claw disease.

The main implication is methodological. Standardized lesion diagnosis should be combined with repeated quarter-level udder-health testing, metabolic profiling, and simultaneous serum and milk sampling. Targeted immunoassays, casein and protease measurements, and LC–MS/MS proteomics or peptidomics could distinguish a true claw-disease-associated response from mastitis, reduced intake, negative energy balance, medication, or lactation stage.

Any raw-milk difference would require separate technological evaluation through standardized processing, with measurements of residual proteins, peptide sequences, coagulation, and proteolysis. Until then, claw-disease-associated changes should not be described as improving or impairing dairy-product bioactivity.

Claw-lesion prevention and treatment clearly benefit welfare and productivity [73,74,75]. Automated lameness detection may facilitate longitudinal study-animal identification [77,78], but molecular milk benefits remain unproven. Herd-level interventions should therefore link hoof-health records with repeated milk APP, protease, proteomic, and peptidomic measurements before welfare improvements are associated with bioactive milk quality.

## 6. Effects of Periparturient Metabolic Disorders on Milk Bioactive Components

### 6.1. Transition-Period Inflammation

Evidence-directness summary: B—systemic for transition-period inflammatory responses, B—milk-omics for broad milk changes associated with energy balance and lactation stage, and C–D for changes in specified bioactive proteins, protease systems, and peptide endpoints attributable to a defined metabolic disorder.

The transition period involves coordinated metabolic, endocrine, and immune adaptation [30,33,35,38,41]. Negative energy balance, NEFA mobilization, hyperketonemia, hypocalcemia, hepatic lipid accumulation, and postpartum inflammation frequently overlap, limiting attribution of a milk protein or peptide change to one disorder.

Milk proteomic and metabolomic studies show broad changes with energy balance and lactation stage [58,59], but do not establish that an isolated transition disorder specifically alters M-Hp, M-SAA, LF, cathelicidins, casein-derived peptides, or other defined bioactive components. Oxidative stress is therefore treated as a modifier of inflammatory, secretory, and proteolytic pathways rather than as a milk bioactive component.

Serum Hp and SAA frequently increase in cows developing postpartum disease, and sustained inflammation is associated with poorer production and reproduction [36,37,38,79]. Direct evidence for milk APP changes caused by systemic transition inflammation independently of intramammary disease remains limited; intramammary challenge models demonstrate mammary responses rather than isolated systemic effects.

‘Excessive’ and ‘prolonged’ inflammation require separate operational definitions because no universal serum Hp or SAA cut-off is transferable across analytical platforms, herds, and postpartum sampling days [37,38]. Excessive inflammation may be defined using assay- and population-specific upper reference limits or prospectively specified high-response categories; for example, serum Hp > 100 µg/mL at one-week postpartum was used to define a high-Hp group in one cohort [79], but this value should not be interpreted as a universal threshold. Longitudinal profiles across the periparturient period are more informative than isolated measurements for identifying a persistent inflammatory response [37,38]. Accordingly, prolonged inflammation may be operationalized in future studies as Hp and/or SAA remaining above the study-specific reference interval at two or more consecutive postpartum sampling points or failing to decline over a prespecified period. Because validated universal duration and concentration thresholds are unavailable, studies should report the assay, sampling days, reference population, and the exact criteria used for both magnitude and persistence.

Early-lactation milk APPs may contribute to multi-disease monitoring, although their lack of organ specificity limits interpretation [16,50,69]. Elevated milk Hp or M-SAA may reflect mammary inflammation, systemic disease, colostral transition, or combinations thereof and should be interpreted with repeated quarter-level SCC, udder-health testing, days in milk, parity, and metabolic biomarkers.

### 6.2. Ketosis and Milk Bioactive Composition

Evidence-directness summary: B—milk-omics for broad disease-defined milk metabolomic changes, because these untargeted data do not directly quantify the specified bioactive protein or peptide endpoints; C–D for ketosis-specific alterations in individual bioactive proteins, protease systems, and defined peptide sequences.

Hyperketonemia is commonly defined as blood BHBA ≥ 1.2 mmol/L in early lactation [40,80]. Disease-defined studies show plasma and milk metabolomic differences in subclinical and clinical ketosis [80], but these do not establish a characteristic change in M-Hp, M-SAA, LF, cathelicidins, plasmin, or defined bioactive peptides.

Milk proteomic patterns also vary with energy balance and lactation stage [58,59], but are influenced by days in milk, diet, yield, dry-period length, and normal early-lactation physiology. They provide contextual evidence rather than proof of a ketosis-specific bioactive protein profile.

Hyperketonemia and elevated NEFA can impair immune-cell function and increase susceptibility to intramammary infection [41,81]. Milk APP or proteolytic changes in ketotic cows may therefore be indirect, and udder health should be confirmed at every sampling point.

Ketosis has not been shown to independently activate the milk plasmin system. A possible metabolic regulation of the milk plasmin system has been proposed, but it has not been validated specifically in ketotic dairy cows [21,54,82]; it should therefore remain a category C hypothesis under the evidence-directness framework.

Verification requires longitudinal measurements of blood BHBA and NEFA; quarter-level SCC and bacteriology; milk APPs and host-defense proteins; plasmin-system components and casein degradation products; and sequence-resolved peptides. Controls should be matched for parity, days in milk, yield, diet, and body condition. Until then, defined protein, protease, and peptide effects remain category C or D under the evidence-directness framework.

### 6.3. Other Periparturient Metabolic Disorders

Evidence-directness summary: B—systemic for systemic physiological and inflammatory effects, and C–D for disease-specific changes in milk bioactive proteins and peptides.

Subclinical hypocalcemia can impair immune-cell function and increase susceptibility to mastitis, metritis, and displaced abomasum [34,83]. These secondary disorders may affect milk APPs or barrier permeability, but direct milk bioactive protein and peptide studies in isolated, mastitis-free hypocalcemia are lacking. Calcium status is therefore an upstream modifier and confounder rather than a confirmed direct determinant.

Fatty liver and impaired hepatic function alter nutrient partitioning, endotoxin handling, and systemic acute-phase responses [32,33,37], yet disease-specific milk proteomic or peptidomic evidence is sparse. Displaced abomasum is similarly confounded by reduced intake, hyperketonemia, inflammation, treatment, and other postpartum disease. No characteristic milk bioactive signature is established for these disorders.

Future studies should define diagnostic groups prospectively, report overlap among metabolic and inflammatory disorders, and combine repeated milk sampling with quarter-level udder-health testing, targeted APP and host-defense assays, casein and plasmin measurements, and proteomics or peptidomics. This would separate disease-specific effects from normal colostral and early-lactation changes.

The main limitation is the shortage of direct milk studies with strict diagnoses and control of concurrent disease. Accordingly, most disorder-specific effects are classified as category C or D under the evidence-directness framework in Table 1.

### 6.4. Nutritional Modulation: Relevance to Milk Bioactive Components

Evidence-directness summary: B—systemic for systemic inflammatory and metabolic responses, and C–D for milk bioactive protein and peptide outcomes.

Plant-derived feed additives are included only as intervention models. A parallel change in systemic inflammation and independently measured milk APPs, host-defense proteins, protease activity, or peptide profiles would strengthen evidence linking systemic inflammation with the milk bioactive fraction.

Curcumin, carnosic acid, cinnamaldehyde, eugenol, and related compounds can modify circulating inflammatory or metabolic indicators [84], but most studies have not measured corresponding milk APPs, LF, cathelicidins, casein integrity, plasmin, or defined peptides. They therefore demonstrate systemic intervention effects rather than improved milk bioactive quality.

Current evidence does not support claims that phytogenic supplementation improves the technological, functional, or consumer-health properties of milk. Future trials should measure blood and milk endpoints concurrently and assess transfer of phytogenic compounds or metabolites, sensory effects, and processing consequences [84,85,86].

## 7. Fate and Functionality of Inflammation-Associated Milk Proteins and Peptides

### 7.1. Impact on Processing Quality and Dairy Product Functionality

Evidence-directness summary: A for directly demonstrated processing consequences of mastitic milk, including bacteriologically characterized subclinical mastitis, and D for comparable effects attributed specifically to isolated lameness or metabolic disease.

Technological consequences are best established for mastitic milk. Increased plasmin and leukocyte-derived proteases accelerate casein degradation and increase the release of endogenous peptide fragments [21,54,55,56,57,87,88]. These changes can impair rennet coagulation, gel formation, cheese-making performance, and shelf-life stability [66,67,89,90,91,92]. In a recent study of bacteriologically characterized milk from clinically healthy cows, major mastitis pathogens were associated with prolonged clotting time and poorer curd and whey quality, although curd yield was not significantly altered [89]. The magnitude and nature of these effects depend on pathogen category, inflammatory severity, SCC, lactation stage, milk composition, and the proportion of affected milk entering the bulk tank.

Mastitis also changes the whey-protein fraction and increases inflammatory and host-defense proteins [19,49,64]. However, the individual technological effects of LF or cathelicidins have not been separated adequately from mastitis-associated proteolysis, altered mineral balance, and reduced casein integrity. Direct studies using disease-relevant concentrations in appropriate dairy matrices are required before specific effects on starter cultures, coagulation, or product quality can be inferred.

Comparable processing effects have not been demonstrated for isolated lameness, ketosis, hypocalcemia, or other metabolic disorders. General systemic, compositional, metabolomic, or transcriptomic differences are insufficient to infer changes in coagulation, cheese yield, fermentation, or shelf life.

### 7.2. Processing Stability and Gastrointestinal Bioaccessibility

A disease-associated change is relevant to consumer exposure or downstream biological functionality only if the altered protein or peptide remains present, structurally intact, and biologically active after processing. Heat can denature, aggregate, or reduce the solubility and activity of immunoglobulins, LF, and other immune proteins, depending on temperature–time conditions, concentration, and food matrix [10].

Short peptides are generally less susceptible to heat-induced unfolding than intact proteins, although they may be generated, modified, aggregated, or degraded during processing and digestion [7,11,12,13,62,93]. Thus, a higher concentration in raw milk does not imply a proportional increase in a finished product, in the gastrointestinal tract, or at the site of biological action.

Microbial and endogenous proteases generate product-specific peptide profiles during fermentation and ripening. Pre-existing casein degradation in mastitic milk may alter the available substrate, but the outcome depends on product type, starter culture, coagulation conditions, ripening time, initial protein composition, and the extent of proteolysis [11,12,13,62,91,93].

Robust studies should compare healthy and diagnosis-defined milk through standardized pasteurization, UHT treatment, fermentation, cheese making, storage, and in vitro digestion. Residual intact proteins, sequence-resolved peptides, bioaccessibility, and functional activity should be measured at physiologically relevant concentrations. Without this experimental chain, processing and digestion remain major discontinuities between bovine disease and consumer exposure.

### 7.3. Three-Tier Evidence Chain and One Health Interpretation

#### 7.3.1. Tier 1—Alteration in Raw Milk

Mastitis directly increases selected APPs and host-defense proteins, disrupts the blood–milk barrier, activates proteases, and reduces casein integrity [14,15,16,18,19,50,51,53,55,56,57,87,94]. APPs are established biomarkers of infection and inflammation, although they are not organ- or disease-specific and must be interpreted together with clinical, microbiological, and milk-level information [69]. SCC-associated LC–MALDI-MS/MS findings also demonstrate increased endogenous peptide release and proteolysis in milk [88].

Selected lameness- and ketosis-classified populations show milk metabolomic or lipidomic differences [70,71,80], but targeted milk protein and peptide evidence remains sparse for isolated claw and metabolic disorders. Lameness studies demonstrate a substantial disease burden and periparturient changes in hoof-support structures [68,95,96,97], whereas experimental ketosis and transition-period studies demonstrate hepatic metabolic-network and systemic oxidative-stress alterations [35,98]. These systemic findings provide biological context but do not establish corresponding changes in the milk bioactive protein or peptide fraction.

#### 7.3.2. Tier 2—Retention During Processing and Digestion

The stability and digestive behavior of bovine milk proteins and peptides have been studied in healthy milk, colostrum, purified systems, and dairy products [7,10,11,12,13,62,91,93], but few studies begin with diagnosis-defined milk. The fraction of disease-associated M-Hp, M-SAA, LF, cathelicidins, or peptide fragments remaining intact, bioaccessible, and active is therefore largely unknown.

#### 7.3.3. Tier 3—Biological Effects in Human Consumers

Purified or isolated milk-derived proteins and peptides, including lactoferricin and lactoferrin, show antimicrobial or antiviral activity in experimental systems [3,43]. However, these studies do not establish biological effects from consuming milk produced during bovine disease. No adequate human intervention evidence links disease-associated changes in milk with beneficial or adverse consumer outcomes; elevated disease-associated proteins should therefore not be presented as improving consumer health.

A One Health perspective remains valuable when the three tiers are kept separate. Mastitis prevention, claw-health management, and transition-cow monitoring benefit animal welfare, production management, and farm economics [73,75,79,99]. Clinical mastitis may also impair reproductive performance, particularly when pathogen-specific episodes occur shortly before or after insemination [86]. These animal-health and production benefits are compatible with a One Health perspective [47], independently of any unproven nutritional effect in consumers.

Integrated health records, SCC, milk APPs, protease assays, sensor-derived data, proteomics, peptidomics, and appropriately validated machine-learning methods may improve disease detection and processing-quality control and support the longitudinal evaluation of downstream functionality [77,78,100,101]. However, improvements in cow health or disease-associated changes in raw milk should not be interpreted as evidence of a more beneficial protein or peptide profile for human consumers unless all three evidence tiers are demonstrated: alteration in raw milk, retention and bioaccessibility after processing and gastrointestinal digestion, and a biological effect in human consumers.

## 8. Conclusions and Future Perspectives

Mastitis produces the most consistent and directly measured changes in the bioactive protein and peptide-related fraction of bovine milk. Intramammary inflammation increases acute-phase and host-defense proteins, disrupts the blood–milk barrier, activates proteolytic systems, and reduces casein integrity. By contrast, evidence for lameness and periparturient metabolic disorders remains dominated by systemic biomarkers, broad milk-composition or omics changes, and mechanistic inference. Disease-specific effects on milk bioactive proteins and peptides therefore require confirmation in studies using clearly defined diagnoses and rigorous control for concurrent mastitis.

Key research gaps include lesion-specific milk proteomics and peptidomics in lame cows without concurrent intramammary infection; prospective comparisons of ketosis, hypocalcemia, fatty liver, displaced abomasum, and other transition disorders; harmonization of analytical methods and detection-limit reporting; and tracking of disease-associated proteins and sequence-resolved peptides through dairy processing, gastrointestinal digestion, and functional testing. Human-health interpretations require completion of all three evidence tiers: alteration in raw milk, retention and bioaccessibility after processing and digestion, and biological effects at physiologically relevant exposure levels. Until this evidence chain is complete, neither beneficial nor adverse consumer-health effects should be inferred. Table 3 summarizes the principal evidence gaps and the study designs recommended to address them.

Future research should prioritize longitudinal, diagnosis-specific cohorts with repeated quarter- and cow-level milk sampling; standardized lesion and metabolic diagnoses; SCC and microbiological testing; and integrated APP, protease, proteomic, and peptidomic analyses. Parallel processing and digestion studies are required to determine which disease-associated changes remain bioaccessible and functional, without conflating cow-level inflammation, raw-milk composition, and human-health effects.

## Figures and Tables

**Table 1:** Approximate concentrations or assay-status descriptions, representative detection approaches reported in the cited literature, and evidence-directness classifications for changes in major bioactive proteins, acute-phase proteins, proteolytic markers, and peptide-related endpoints in bovine milk.

Bioactive Component	Detection Approaches Reported in Cited Studies	Healthy Mature Milk	Subclinical Mastitis	Clinical Mastitis	Lameness/Claw Disease	Metabolic Disease
Haptoglobin (M-Hp)	ELISA; immunoturbidimetry [14,15,16,50,51]	Low or near the assay-specific detection limit in healthy control milk; no universal reference interval has been established [14,15].	A: increased [14,15,16,50].	A: markedly increased [14,15,16,50,51].	B—systemic: serum Hp may increase in severe claw lesions, but a lesion-specific increase in milk has not been demonstrated [26,52].	C: an increase in milk during systemic postpartum inflammation is plausible, but direct disorder-specific validation is lacking [14,15,16,30,31,32,37,38,41].
Milk serum amyloid A/mammary-associated serum amyloid A3 (M-SAA/M-SAA3)	ELISA; immunoassay; immunoblot [15,16,51]	Low and assay-dependent in healthy mature milk; no universal reference interval has been established [15,16].	A: increased [15,16].	A: markedly increased [15,16,51].	B—systemic: systemic SAA responses occur in selected lesions, but direct lesion-specific milk M-SAA/M-SAA3 evidence is lacking [26,52].	C: a milk-associated increase during postpartum inflammation is plausible, but direct disorder-specific validation is lacking [15,16,30,31,32,37,38,41].
Lactoferrin (LF)	ELISA [48]; radial immunodiffusion [3]; LC–MS/MS [16]	Approximately 0.03–0.5 mg/mL in healthy mature individual-cow or quarter milk; strongly dependent on lactation stage, SCC, sampling design, and analytical method [3,48].	A: increased, although the magnitude varies with pathogen, SCC, and lactation stage [3,16,48].	A: increased, with larger responses generally associated with more severe mammary inflammation [3,16,48].	D: evidence gap; no direct disease-specific milk evidence or sufficiently supported endpoint-specific mechanism was identified.	D: evidence gap; no direct disease-specific milk evidence or sufficiently supported endpoint-specific mechanism was identified.
Lipopolysaccharide-binding protein (LBP)	ELISA; targeted proteomics [16,49]	Detectable at low µg/mL concentrations; assay- and study-dependent [16,49].	A: milk concentrations differ between uninfected and subclinically infected quarters in direct studies [16,49].	A: increased in naturally acquired clinical mastitis and after intramammary LPS challenge [16,49].	D: evidence gap; no direct disease-specific milk evidence or sufficiently supported endpoint-specific mechanism was identified.	C: an increase during systemic inflammation is mechanistically plausible, but disease-specific milk validation is lacking [16,30,31,32,37,38,39,41,49].
α1-Acid glycoprotein (AGP)	Western blotting; radial immunodiffusion; anion-exchange enrichment; immunoassay [16,51]	Below the detection limit of radial immunodiffusion in mature milk, but detectable using enrichment-based or more sensitive methods [16,51].	C: a disease-associated milk change is plausible, but robust targeted quantitative evidence is insufficient [16,19,51].	C: direct quantitative clinical-mastitis-specific milk validation is insufficient [16,19,51].	D: evidence gap; no direct disease-specific milk evidence or sufficiently supported endpoint-specific mechanism was identified.	C: a disease-associated milk change is plausible, but direct disorder-specific evidence is lacking [30,31,32,37,38,41].
Cathelicidins	Targeted LC–MS/MS; immunoblot; ELISA [16,50,53]	Not detected or present at very low abundance in healthy quarters in the cited immunoblot studies; assay-dependent [16].	A: increased [16,50,53].	A: increased [16,50].	D: evidence gap; no direct disease-specific milk evidence or sufficiently supported endpoint-specific mechanism was identified.	D: evidence gap; no direct disease-specific milk evidence or sufficiently supported endpoint-specific mechanism was identified.
Lysozyme	Enzymatic lysis or activity assay; immunoassay [16]	Low and assay-dependent; approximately 6.9 µg/mL as reported in the reviewed literature [16]	A: increased in a direct study of staphylococcal mastitis; broader validation is limited [16].	A: increased in a direct study of staphylococcal mastitis; broader validation is limited [16].	D: evidence gap; no direct disease-specific milk evidence or sufficiently supported endpoint-specific mechanism was identified.	D: evidence gap; no direct disease-specific milk evidence or sufficiently supported endpoint-specific mechanism was identified.
Immunoglobulins, especially IgG	ELISA [8]; radial immunodiffusion [2,48]	Approximately 0.2–0.5 mg/mL for IgG1 or total IgG in representative mature quarter-milk studies; strongly dependent on isotype, lactation stage, SCC, sampling unit, and assay [2,8,48].	A: increased in selected direct studies, consistent with blood–milk barrier leakage [18,19,20,48].	A: increased, consistent with pronounced barrier disruption [18,19,20].	C: a barrier-mediated change is plausible but unconfirmed [18,20,26,52].	C: a disease-specific milk change remains unconfirmed and is strongly confounded by colostral transition and concurrent mammary inflammation [18,20,30,31,32,37,38,41].
α- and β-casein integrity	SDS–PAGE; HPLC; LC–MS/MS [21,54,55,56]	Intact reference profile [21,54].	A: decreased through increased proteolysis [21,54,55,56].	A: markedly decreased through proteolysis [21,54,55,57].	C: systemic inflammatory or secondary disease-related effects are plausible but unconfirmed [21,26,52].	C: disease-specific changes in α- and β-casein integrity are plausible, but direct validation in cows with a defined metabolic disorder is lacking. Broad transition-period milk-proteomic findings provide contextual rather than disorder-specific evidence [58,59].
Plasmin activity in milk	Chromogenic or fluorometric activity assay [17,21,54,55,56]	Basal activity is present and regulated by the balance among plasminogen, plasminogen activators, and inhibitors; values are assay-dependent [17,21].	A: increased [17,21,56].	A: increased [17,21,54,55].	C: a change is plausible but has not been established for isolated claw disease [17,21,26,52].	C: metabolic regulation is hypothesized, but direct disease-specific milk validation is required [21,54,58,59].
ACE-inhibitory peptides (VPP, IPP)	Targeted LC–MS/MS; peptidomics; ACE-inhibition assay [7]	No established concentration range for free, sequence-resolved peptides in raw milk; the relevant sequences are primarily encrypted within caseins and may be released during processing or digestion [4,5].	C: altered substrate integrity may affect later peptide release, but direct sequence-resolved evidence is limited [4,5,21,54,55,56].	C: extensive proteolysis may alter later peptide availability, but direct evidence is limited [4,5,21,54,55,56,57].	D: evidence gap; no direct disease-specific milk evidence or sufficiently supported endpoint-specific mechanism was identified.	C: altered casein integrity or protease regulation may affect the subsequent release of VPP and IPP during processing or digestion, but no disease-specific sequence-resolved evidence is available [4,5,21,54,58,59].
β-Casomorphin-7 (BCM-7)	Targeted LC–MS/MS; peptidomics [7,60,61,62]	No established concentration range for free BCM-7 in raw milk; release depends mainly on β-casein genotype and digestion [60,61,62].	C: altered β-casein integrity may affect subsequent release, but this has not been demonstrated [21,54,55,56,60,61,62].	C: mechanistically plausible, but direct disease-defined evidence is lacking [21,54,55,56,57,60,61,62].	D: evidence gap; no direct disease-specific milk evidence or sufficiently supported endpoint-specific mechanism was identified.	C: altered β-casein integrity may affect subsequent BCM-7 release during processing or digestion, but no disease-specific sequence-resolved evidence is available [21,54,58,59,60,61,62].

Note: Changes are expressed relative to healthy mature milk. Evidence-directness categories were assigned as follows: A, direct milk-level evidence for the specified protein, protease, peptide, or explicitly defined milk endpoint from animals with a defined diagnosis or from experimental mammary-challenge studies; B, relevant evidence that does not directly quantify the specified endpoint; C, a specific mechanistic relationship supported by relevant indirect or non-disease-specific evidence but requiring disease-specific milk validation; and D, neither direct disease-specific milk evidence nor a sufficiently specific, evidence-supported mechanistic relationship identified. Category D indicates an evidence gap and should not be interpreted as evidence that no disease-associated change occurs. These categories describe evidence directness and endpoint specificity only and do not represent a formal assessment of study quality, sample size, risk of bias, consistency, reproducibility, or overall certainty. The terms “low”, “undetectable”, and “near LOQ” are assay-dependent and do not indicate biological absence or constitute universal reference intervals. Detection approaches in the second column are representative methods reported in the cited literature for each analyte; they do not imply that every cited study used every listed method. Where method-specific citations are shown in the second column, they identify the cited sources supporting the listed analytical approach. Values obtained using ELISA, immunoturbidimetry, electrophoresis, activity assays, and mass-spectrometry-based methods should not be compared directly without considering analytical sensitivity, specificity, calibration, sample preparation, and analyte coverage. The metabolic-disease column excludes normal physiological colostral changes except where these constitute an important confounder. M-SAA is used where the assay did not resolve the isoform; M-SAA3 is used only for isoform-specific measurements. ACE, angiotensin-converting enzyme; AGP, α1-acid glycoprotein; BCM-7, β-casomorphin-7; ELISA, enzyme-linked immunosorbent assay; HPLC, high-performance liquid chromatography; IgG, immunoglobulin G; IPP, Ile-Pro-Pro; LBP, lipopolysaccharide-binding protein; LC–MS/MS, liquid chromatography–tandem mass spectrometry; LOQ, limit of quantification; M-Hp, milk haptoglobin; M-SAA, milk serum amyloid A when the assay does not resolve the isoform; M-SAA3, mammary-associated serum amyloid A3; SDS–PAGE, sodium dodecyl sulfate–polyacrylamide gel electrophoresis; VPP, Val-Pro-Pro.

**Table 3:** Evidence gaps and recommended study designs for investigating inflammation-associated changes in the bioactive protein and peptide profile of bovine milk.

Research Area	Current Evidence	Main Limitation	Recommended Study Design
Mastitis and intramammary infection	Best-documented category. Subclinical and clinical mastitis increase milk APPs (Hp, SAA), host-defense proteins, blood–milk barrier permeability, SCC, and endogenous protease activity [14,15,16,18,19,22,49,64]. SCC-associated peptidomic evidence additionally demonstrates increased generation of casein-derived fragments, without necessarily identifying increased concentrations of defined bioactive peptide sequences [87,88].	Heterogeneity related to pathogen, lactation stage, SCC threshold, quarter-level versus cow-level sampling, analytical method, and disease severity.	Longitudinal quarter-level studies combining pathogen identification, SCC, milk APPs, lactoferrin, cathelicidins, plasmin and MMP activity, proteomics, peptidomics, and processing-quality testing.
Lameness and claw disorders	Lesion-associated systemic acute-phase responses are documented, and selected lameness-classified cohorts show broad milk metabolomic or lipidomic differences [26,27,70,71]. However, these findings do not directly quantify lesion-specific milk bioactive proteins, protease systems, or defined peptide sequences. Direct targeted evidence for changes in the milk bioactive protein and peptide fraction remains sparse.	Few studies quantify milk bioactive proteins in cows with well-characterized claw lesions while controlling for concurrent mastitis, parity, lactation stage, metabolic status, feed intake, and treatment.	Prospective, claw-lesion-specific longitudinal cohort studies using locomotion scoring; diagnosis during professional claw trimming; repeated quarter-level SCC measurements; bacteriological culture or PCR to exclude concurrent intramammary infection; serum APP assays; and targeted milk proteomics and peptidomics.
Ketosis and other periparturient metabolic disorders	Systemic and hepatic evidence is substantial, including ketosis-associated alterations in hepatic metabolic and signaling gene networks and transition-period changes in oxidative status [35,98]. However, direct disease-specific evidence for individual milk proteins, protease systems, and peptide sequences remains limited [40,58,59,80]. Ketosis has the most data; hypocalcemia, fatty liver, and displaced abomasum remain poorly characterized at the targeted milk-protein and peptide level.	Confounding by early lactation, parity, diet, milk yield, concurrent mastitis, and overlap among metabolic disorders.	Prospective longitudinal studies with predefined diagnostic criteria and explicit reporting of disease overlap; repeated SCC and bacteriological testing; and parallel measurement of BHBA, NEFA, calcium, liver indicators, milk APPs, protease activity, proteomics, and peptidomics.
Processing and dairy-product functionality	Mastitis-associated proteolysis impairs casein integrity, rennet coagulation, gel formation, cheese-making performance, and shelf-life stability [21,54,55,56,66,67,89,90,91,92].	Most studies do not link animal-health status, milk bioactive proteins, protease activity, and technological outcomes within the same experimental design.	Integrated studies linking diagnosis-specific animal-health data and raw-milk proteomic profiles with standardized processing conditions, rennet-coagulation properties, cheese yield, shelf-life stability, and sequence-resolved peptide profiles.
Human-health relevance	No direct human-health evidence links disease-associated milk changes with consumer outcomes. Upstream evidence demonstrates altered protein concentrations and proteolysis in raw mastitic milk [14,15,16,19,64], whereas separate experimental studies report biological activities of purified proteins, peptides, or conventional dairy products [1,4,5,6,9,43,44]. Few studies have tracked disease-associated changes through processing and digestion.	Evidence is discontinuous across three tiers: alteration in raw milk, retention and bioaccessibility after processing and digestion, and biological effects in human consumers. Detectability or activity in an isolated experimental system does not establish consumer exposure or health effects.	Studies beginning with milk from cows of clearly defined health status, followed by standardized processing, simulated gastrointestinal digestion, quantitative protein and peptide analysis, physiologically relevant cell-based assays, microbiome-interaction assays, and, only where justified, human intervention studies.
Precision livestock farming and herd-level monitoring	Automated SCC, lameness, metabolic, and real-time milk-composition monitoring can support diagnosis-specific animal selection and longitudinal sampling [77,78,100,101]. These tools do not directly establish changes in milk bioactive proteins or peptides.	Lack of large-scale herd-level datasets linking automated health records with validated milk protein and peptide biomarkers and downstream processing outcomes.	Large-scale longitudinal herd studies integrating automated milking and sensor data, disease records, repeated milk sampling, validated biomarker panels, proteomics and peptidomics, and dairy-processing endpoints.

APP = acute-phase protein; BHBA = β-hydroxybutyrate; Hp = haptoglobin; MMP = matrix metalloproteinase; NEFAs = non-esterified fatty acids; PCR = polymerase chain reaction; SAA = serum amyloid A; SCC = somatic cell count. This table distinguishes areas with strong direct milk-level evidence, particularly mastitis, from areas in which current knowledge is based mainly on systemic inflammatory evidence, broad omics findings, mechanistic plausibility, or limited disease-specific milk data.

## Data Availability

No new data were created or analyzed in this study. Data sharing is not applicable to this article.

## Abbreviations

The following abbreviations are used in this manuscript:

Abbreviation	Definition
ACE	angiotensin-converting enzyme
AGP	α1-acid glycoprotein
APP	acute-phase protein
BCM-7	β-casomorphin-7
BHBA	β-hydroxybutyrate
BSA	bovine serum albumin
*CSN2*	β-casein-encoding gene
ELISA	enzyme-linked immunosorbent assay
HPLC	high-performance liquid chromatography
Hp	haptoglobin
IgG	immunoglobulin G
IL-1β	interleukin-1β
IL-6	interleukin-6
IPP	Ile-Pro-Pro
LBP	lipopolysaccharide-binding protein
LC–MS/MS	liquid chromatography–tandem mass spectrometry
LC–MALDI-MS/MS	liquid chromatography–matrix-assisted laser desorption/ionization tandem mass spectrometry
LF	lactoferrin
LOQ	limit of quantification
LPS	lipopolysaccharide
MEC	mammary epithelial cell
MEDLINE	Medical Literature Analysis and Retrieval System Online
M-Hp	milk haptoglobin
M-SAA	milk serum amyloid A when the assay does not resolve the isoform
M-SAA3	mammary-associated serum amyloid A3
MMP	matrix metalloproteinase
MMP-2	matrix metalloproteinase-2
MMP-9	matrix metalloproteinase-9
NEFA	non-esterified fatty acid
PA	plasminogen activator
PCR	polymerase chain reaction
PG	phosphatidylglycerol
Plg	plasminogen
SAA	serum amyloid A
SCC	somatic cell count
SCM	subclinical mastitis
SDS–PAGE	sodium dodecyl sulfate–polyacrylamide gel electrophoresis
TJ	tight junction
TNF-α	tumor necrosis factor-α
UHT	ultra-high-temperature treatment
VPP	Val-Pro-Pro
